# Using pre-selected variants from large-scale whole-genome sequence data for single-step genomic predictions in pigs

**DOI:** 10.1186/s12711-023-00831-0

**Published:** 2023-07-26

**Authors:** Sungbong Jang, Roger Ros-Freixedes, John M. Hickey, Ching-Yi Chen, Justin Holl, William O. Herring, Ignacy Misztal, Daniela Lourenco

**Affiliations:** 1 Department of Animal and Dairy Science, University of Georgia, Athens, GA 30602 USA; 2 Departament de Ciència Animal, Universitat de Lleida-Agrotecnio-CERCA Center, Lleida, Spain; 3 The Roslin Institute and Royal (Dick) School of Veterinary Studies, The University of Edinburgh, Easter Bush, Midlothian, Scotland UK; 4 The Pig Improvement Company, Genus Plc, Hendersonville, TN USA

## Abstract

**Background:**

Whole-genome sequence (WGS) data harbor causative variants that may not be present in standard single nucleotide polymorphism (SNP) chip data. The objective of this study was to investigate the impact of using preselected variants from WGS for single-step genomic predictions in maternal and terminal pig lines with up to 1.8k sequenced and 104k sequence imputed animals per line.

**Methods:**

Two maternal and four terminal lines were investigated for eight and seven traits, respectively. The number of sequenced animals ranged from 1365 to 1491 for the maternal lines and 381 to 1865 for the terminal lines. Imputation to sequence occurred within each line for 66k to 76k animals for the maternal lines and 29k to 104k animals for the terminal lines. Two preselected SNP sets were generated based on a genome-wide association study (GWAS). Top40k included the SNPs with the lowest p-value in each of the 40k genomic windows, and ChipPlusSign included significant variants integrated into the porcine SNP chip used for routine genotyping. We compared the performance of single-step genomic predictions between using preselected SNP sets assuming equal or different variances and the standard porcine SNP chip.

**Results:**

In the maternal lines, ChipPlusSign and Top40k showed an average increase in accuracy of 0.6 and 4.9%, respectively, compared to the regular porcine SNP chip. The greatest increase was obtained with Top40k, particularly for fertility traits, for which the initial accuracy based on the standard SNP chip was low. However, in the terminal lines, Top40k resulted in an average loss of accuracy of 1%. ChipPlusSign provided a positive, although small, gain in accuracy (0.9%). Assigning different variances for the SNPs slightly improved accuracies when using variances obtained from BayesR. However, increases were inconsistent across the lines and traits.

**Conclusions:**

The benefit of using sequence data depends on the line, the size of the genotyped population, and how the WGS variants are preselected. When WGS data are available on hundreds of thousands of animals, using sequence data presents an advantage but this remains limited in pigs.

**Supplementary Information:**

The online version contains supplementary material available at 10.1186/s12711-023-00831-0.

## Background

Using single nucleotide polymorphism (SNP) chip data for genomic prediction relies on the linkage disequilibrium (LD) between SNPs and causative variants [1]. Because of the initial high cost of SNP genotyping, most of the SNP chips used in livestock are still limited to less than 100k SNPs, which could restrict the information available for genomic predictions. Whole-genome sequence (WGS) data harbor millions of variants, possibly including causative variants that primarily affect the traits of interest but are not present on regular SNP chips. With the decreasing sequencing costs, the availability of WGS data for some agricultural species is increasing. Whether such data can help increase the accuracy of genomic predictions beyond that already achieved by SNP chips is still questionable since marginal or no gains were reported by several studies [2–5]. Specifically, in pigs, Zhang et al. [6] showed that the 80k SNP chip outperformed the 650k SNP chip and WGS data for genomic predictions of average daily feed intake and backfat traits. In contrast, Song et al. [7] reported a marginal gain in prediction accuracy when WGS data were used. The absence of benefits reported in those studies could be due to the small number of sequenced animals (maximum of 289 animals), poor imputation accuracy, the statistical methods used, and to sequenced SNPs being redundant with those already included on the standard chip data. The largest study on genomic prediction using WGS data in pigs, conducted by Ros-Freixedes et al. [8], examined nearly 400k pigs from seven lines with imputed WGS data. Compared to the use of regular SNP chips, they found small improvements in prediction accuracy, averaging at 0.025, for eight common complex traits. These results highlighted the need for large datasets and for optimized pipelines to exploit WGS data.

Imputation is an inevitable step when working with WGS data because sequencing many individuals is still not feasible. Currently, the most efficient approach is to sequence a subset of the animals in a population and impute the sequence data to other animals that are already genotyped with SNP arrays [9]. Using all variants from WGS data may not benefit genomic predictions since they may not all be causative or in high LD with the causative variants [10]. Hence, preselection of variants helps narrow down the WGS data to the significant ones only. Previous studies have explored several methods to identify significant or causative variants for genomic prediction, such as genome-wide association studies (GWAS) [3], SNP functional annotation [11], and gene expression [12]. Among these approaches, GWAS has been commonly used to preselect WGS variants in pig populations [6–8].

Fragomeni et al. [13] used simulated sequence data and, on the one hand, demonstrated that the prediction accuracy could be optimized once the position of all causative variants and the percentage of additive variance they explain are known. On the other hand, the same authors reported that the accuracy is inversely related to the distance between the causative variants and the neighboring SNPs if only the neighboring SNPs are identified. Although it is theoretically plausible to enhance prediction accuracy by using causative variants for genomic prediction, the use of real data has only shown small or no improvements from genomic prediction [2, 14, 15]. Gualdrón-Duarte et al. [14] indicated an increase in prediction accuracy of up to seven points for carcass traits when using single-step genomic best linear unbiased prediction (ssGBLUP) with BayesR SNP weights on several known causative variants from real beef cattle data. However, no improvements were observed with non-linear weights [2, 16]. Liu et al. [15] also observed non-significant benefits from weighted ssGBLUP (WssGBLUP) in dairy cattle, where the weighted matrix was constructed from a Bayesian whole-genome regression model (i.e., BayesN).

Using simulated sequence data, Jang et al. [17] investigated the dimensionality of the genomic information [18] to assess the number of genotyped animals required to optimize the percentage of variant discoveries in GWAS. They showed that using a number of genotyped animals that is equal to the number of eigenvalues that explain 98% of the variance of the genomic relationship matrix is sufficient to capture causative variants in populations with a larger effective size (*Ne* = 200). In contrast, populations with a small effective size (*Ne* = 20) require more genotyped animals. Although *Ne* plays a role, discovering causative variants remains difficult if the genotyped animals have a limited number of progeny with records.

In pigs, the *Ne* of typical commercial breeding populations ranges from 30 to 50 and the dimensionality of the genomic information or the number of independent chromosome segments (*Me*) that segregate in a population ranges from 4000 to 6000 [19]. Based on Jang et al. [17], using a sample size of 7000 for GWAS in a population with a *Ne* of 20 allowed the detection of causative variants that explain 20% of the additive genetic variance. Moreover, larger sample sizes resulted in better prediction accuracies with variants selected from GWAS. Recently, Ros-Freixedes et al. [8] and Ros-Freixedes et al. [20] proposed an approach to generate accurate imputed WGS data for hundreds of thousands of pigs across multiple lines and assessed the suitability of WGS variants that were preselected using GWAS-based methods for genomic prediction using BayesR [21, 22]. However, BayesR only considers data from genotyped animals. In most livestock populations, only a small fraction of the phenotyped animals is genotyped. In such a situation, single-step methods (i.e., ssGBLUP [23–25] or ssSNP-BLUP [26]) are advantageous because they also incorporate information on non-genotyped individuals into the analysis. In addition, most breeding programs currently use single-step methods [23, 27–29]. Therefore, in this study we investigated the impact of using preselected variants from WGS data for genomic prediction with ssGBLUP in maternal and terminal pig lines, with up to 1800 sequenced and 104,000 imputed sequenced animals per line. We explored different sets of preselected variants and the changes in accuracy when using ssGBLUP and WssGBLUP with BayesR SNP variances as weights.

## Methods

### Data

Datasets provided by the Pig Improvement Company (PIC; Hendersonville, TN) comprised two maternal lines (ML1 and ML2) and four terminal lines (TL1, TL2, TL3, and TL4) with diverse genetic backgrounds, for which the breeds of origin were Landrace and Large White for the maternal lines, and Duroc, Hampshire, and Large White for the terminal lines. For the maternal lines, we analyzed average daily feed intake (ADFI), average daily gain (ADG), backfat thickness (BF), loin depth (LDP), total number of piglets born (TNB), number of stillborn (NSB), return to oestrus seven days after weaning (RET), and litter weaning weight (WWT). For the terminal lines, we analyzed ADFI in the purebreds, and ADG, BF, and LDP in both purebreds and crossbreds (ADGX, BFX, and LDPX). The total number of animals in the pedigree and records for each trait are in Table 1.

**Table 1:** Number of records and animals in the pedigree

Line	ADFI	ADG	BF	LDP	TNB	NSB	RET	WWT	Pedigree
ML1	35k	1.06M	820k	604k	1.08M	1.13M	0.86M	34k	3.75M
ML2	34k	1.52M	936k	631k	5.11M	5.28M	4.10M	29k	9.18M

	ADFI	ADG	BF	ADGX	BFX	LDP	LDPX	Pedigree
TL1	35k	356k	339k	150k	149k	305k	148k	1.13M
TL2	40k	298k	295k	158k	156k	294k	156k	0.84M
TL3	16k	233k	226k	155k	153k	212k	152k	1.30M
TL4	64k	937k	859k	299k	247k	753k	243k	3.14M

ADFI: average daily feed intake; ADG: average daily gain; BF: backfat thickness; LDP: loin depth; TNB: total number of piglets born; NSB: number of stillborn; RET: return to oestrus seven days after weaning; WWT: litter weaning weight; ADGX: ADG recorded in crossbred; BFX: BF recorded in crossbreds; LDPX: LDP recorded in crossbreds; ML1: maternal line 1; ML2: maternal line 2; TL1: terminal line 1; TL2: terminal line 2; TL3: terminal line 3; TL4: terminal line 4

Some traits were jointly analyzed in multi-trait models for genomic prediction. For the maternal lines, two-trait models were considered for ADG and ADFI (ADFI model), ADG, and BF (GROWTH model), ADG and LDP (LOIN model), and TNB and NSB (REPROD model), but single-trait models were used for RET (RET model) and WWT (WWT model). For the terminal lines, the ADFI model used for the maternal lines was also applied, but four-trait models were used for the GROWTH (ADG, BF, ADGX, and BFX) and the LOIN (ADG, LDP, ADGX, and LDPX) models.

Pigs were initially genotyped with either the GGP-Porcine LD BeadChip or the HD BeadChip (GeneSeek, Lincoln, NE) and then were imputed up to 50k. In each line, we filtered out SNPs that were monomorphic, with a call rate lower than 0.90, a minor allele frequency lower than 0.01, and a difference between observed and expected genotype frequencies greater than 0.15. We also removed the individuals with more than 10% missing genotypes. Table 2 shows the number of genotyped animals and SNPs by line after quality control.

**Table 2:** Number of genotyped individuals, SNPs, sequenced, and imputed sequenced animals in the two maternal and four terminal lines

Line	Number of genotyped individuals	Number of SNPs (chip)	Number of sequenced individuals	Number of imputed sequenced individuals
ML1	76,227	40,592	1366	76,230
ML2	66,608	42,746	1491	66,608
TL1	60,467	35,786	731	60,474
TL2	41,572	40,311	760	41,573
TL3	29,328	39,999	381	29,330
TL4	104,644	43,032	1856	104,661

ML1: maternal line 1; ML2: maternal line 2; TL1: terminal line 1; TL2: terminal line2; TL3: terminal line 3; TL4: terminal line 4; Chip: 50k chip data

### Whole-genome sequencing and imputation

The WGS data used in this study were generated by Ros-Freixedes et al. [8] and Ros-Freixedes et al. [20]. In summary, a low-coverage sequencing strategy was followed by joint calling, phasing, and imputation of the WGS genotypes using the ‘hybrid peeling’ method implemented in AlphaPeel [30]. Table 2 shows the number of individuals sequenced and imputed to sequence for each line. The ‘hybrid peeling’ method used genotypes from both marker arrays (GGP-Porcine LD and HD) and the WGS data that are available across the complete multi-generational pedigrees. Imputation was carried out separately for each line. Individuals were predicted to have a low imputation accuracy if they or their grandparents were not genotyped with a marker array or if they were less connected (based on the sum of coefficients of pedigree relationships) to the rest of the population, as described in Ros-Freixedes et al. [20], and were excluded [20]. The number of imputed individuals that remained for each line after quality control is in Table 2. Based on the imputation accuracy of 284 pigs that had both WGS (high coverage) and marker array data, these individuals were predicted to have an average dosage correlation of 0.97 (median: 0.98), defined as the individual-wise correlation between true and imputed genotypes [20]. All SNPs with a minor allele frequency lower than 0.023 were removed since their estimated dosage correlations were less than 0.90 [20]. After imputation, genotypes (i.e., 0/1/2) were called for all individuals, including those that were directly sequenced.

### Training and test sets

Before the GWAS, all animals with WGS data were separated into training and test sets, as defined in Ros-Freixedes et al. [8]. Test sets were generated by extracting entire litters from the last generation of the pedigree and only considering litters with a minimum of five full sibs. All remaining WGS individuals were considered as training sets. The training sets were filtered by excluding individuals whose relationship coefficient with individuals in the test sets was equal to or greater than 0.5. This ensured that the improvement in prediction accuracy was not solely attributed to the close relatedness between individuals in the training and test sets but to the information gained from the WGS data. This approach was also intended to mimic a practical pig breeding scheme where selection candidates available in a selection nucleus at a specific time are evaluated [8]. The same training set was used for GWAS and genomic predictions in each line. Previous studies reported reduced prediction accuracy and bias of genomic estimated breeding value (GEBV) when using the same dataset for GWAS and genomic prediction [31, 32]. However, Ros-Freixedes et al. [8] conducted a study using the same data as ours and they found no systematic changes in accuracy and dispersion after splitting the training set into two exclusive subsets, one for GWAS and one for genomic prediction.

### Pre-selected SNP panels

Two different pre-selected SNP panels were created based on the WGS data for genomic prediction, as described in Ros-Freixedes et al. [8]: (1) Top40k and (2) ChipPlusSign. Top40k refers to a set of variants with the lowest p-values, identified through GWAS in each consecutive non-overlapping 55-kb window across the genome, where each window was of equal size. Notably, these variants were not necessarily below the significance threshold but were selected based on their p-values and location within each window. ChipPlusSign combined the 50k chip data (Chip) and significant variants (p ≤ 10^–6^), based on a significance threshold of 0.05 that accounts for multiple-testing using the Bonferroni correction. We assumed that the markers from the Chip (~ 40k) were independent. When multiple significant variants were within a 55-kb window, only the variant with the lowest p-value was selected. We used a univariate linear mixed model for single-SNP GWAS within each trait and line through the FastLMM software [33], by including only the genotyped individuals from the training set. Population structure was accounted for by the genomic relationship matrix ($\mathbf{G}$). More details about the GWAS are reported by Ros-Freixedes et al. [8]. Genomic predictions with the two sets were compared against prediction based on the Chip (Table 2). We combined each pre-selected variant set for the traits included in each model if the scenarios used multi-trait models. For example, the pre-selected variants for ADFI and for ADG were combined for the ADFI model and used for genomic prediction. As WGS information was available only on purebred animals, no GWAS variants were selected for ADGX, BFX, and LDPX. All the combinations of selected variants for each model are described in Additional file 1: Table S1. After constructing all the pre-selected SNP panels, a quality control step was applied to remove SNPs with a difference between observed and expected genotype frequencies greater than 0.15 and to exclude individuals with parent-progeny Mendelian conflicts. Additional file 1: Tables S2 and S3 show the numbers of animals and SNPs for all pre-selected SNP panels after quality control for the maternal and terminal lines. Because the number of animals available for each preselected SNP panel (Top40k and ChipPlusSign) and Chip was different after quality control, the training and test sets contained only animals that passed quality control for all SNP panels (see Additional file 1: Table S4). This step guaranteed a fair comparison of genomic predictions between scenarios (Table 3).

**Table 3:** Number of animals with genomic information that were retained after quality control and used in the analyses with all the SNPs panels

Line	ADFI	GROWTH	LOIN	REPROD	RET	WWT
ML1	74,148	74,153	74,152	73,919	73,891	74,058
ML2	64,654	64,655	64,659	64,599	64,653	63,456
TL1	56,423	56,424	56,422	–	–	–
TL2	38,477	38,475	38,477	–	–	–
TL3	27,671	27,671	27,671	–	–	–
TL4	102,586	102,590	102,588	–	–	–

For maternal lines ML1 and ML2; ADFI: two-trait ADFI model (ADG and ADFI); GROWTH: two-trait GROWTH model (ADG and BF); LOIN: two-trait LOIN model (ADG and LDP); REPROD: two-trait REPROD model (TNB and NSB); RET: single-trait RET model (RET); WWT: single-trait WWT model (WWT)

For terminal lines TL1, TL2, TL3, and TL4; ADFI: two-trait GROWTH model (ADG and ADFI); GROWTH: four-trait GROWTH model (ADG, BF, ADGX, and BFX); LOIN: four-trait LOIN model (ADG, LDP, ADGX, and LDPX)

### Genomic prediction

Single-trait, two-trait, or four-trait models were used for genomic prediction, depending on the traits. Here, only the four-trait GROWTH model (ADG, BF, ADGX, and BFX) of terminal lines is described:

$$\mathbf{y}=\mathbf{X}\mathbf{b}+\mathbf{W}\mathbf{c}+\mathbf{Z}\mathbf{u}+\mathbf{e},$$

where $\mathbf{y}$ is the vector of phenotypes; $\mathbf{X}$ is an incidence matrix for fixed effects (contemporary group as a cross-classified effect for all traits, off-test weight and carcass weight as a covariate only for BF and BFX, respectively) contained in $\mathbf{b}$; $\mathbf{W}$ is an incidence matrix for the random, diagonal litter effect contained in $\mathbf{c}$ ($\mathbf{c}\sim \mathrm{MVN}(0,\mathbf{I}\otimes{\mathbf{L}}_{0}$)); $\mathbf{Z}$ is an incidence matrix for the random additive genetic effect contained in $\mathbf{u}$ ($\mathbf{u}\sim \mathrm{MVN}(0,\mathbf{H}\otimes{{\varvec{\Sigma}}}_{0}$)); and $\mathbf{e}$ ($\mathbf{e}\sim \mathrm{MVN}(0,\mathbf{I}\otimes{\mathbf{R}}_{0}$)) is a vector of residual effects. Matrices ${\mathbf{L}}_{0}$, ${\varvec{\Sigma}}$_0_, and ${\mathbf{R}}_{0}$ are as follows:

$${\mathbf{L}}_{0}=\left[\begin{array}{cccc}{\sigma }_{{l}_{ADG}}^{2}& {\sigma }_{{l}_{ADG,}{l}_{BF}}& 0& 0\\ {\sigma }_{{l}_{BF,}{l}_{ADG}}& {\sigma }_{{l}_{BF}}^{2}& 0& 0\\ 0& 0& {\sigma }_{{l}_{ADGX}}^{2}& {\sigma }_{{l}_{ADGX,}{l}_{BFX}}\\ 0& 0& {\sigma }_{{l}_{BFX,}{l}_{ADGX}}& {\sigma }_{{l}_{BFX}}^{2}\end{array}\right],$$

$${{\varvec{\Sigma}}}_{0}=\left[\begin{array}{cccc}{\sigma }_{{a}_{ADG}}^{2}& {\sigma }_{{a}_{ADG,}{a}_{BF}}& {\sigma }_{{a}_{ADG,}{a}_{ADGX}}& {\sigma }_{{a}_{ADG,}{a}_{BFX}}\\ {\sigma }_{{a}_{BF,}{a}_{ADG}}& {\sigma }_{{a}_{BF}}^{2}& {\sigma }_{{a}_{BF,}{a}_{ADGX}}& {\sigma }_{{a}_{BF,}{a}_{BFX}}\\ {\sigma }_{{a}_{ADGX,}{a}_{ADG}}& {\sigma }_{{a}_{ADGX,}{a}_{BF}}& {\sigma }_{{a}_{ADGX}}^{2}& {\sigma }_{{a}_{ADGX,}{a}_{BFX}}\\ {\sigma }_{{a}_{BFX,}{a}_{ADG}}& {\sigma }_{{a}_{BFX,}{a}_{BF}}& {\sigma }_{{a}_{BFX,}{a}_{ADGX}}& {\sigma }_{{a}_{BFX}}^{2}\end{array}\right],$$

$${\mathbf{R}}_{0}=\left[\begin{array}{cccc}{\sigma }_{{e}_{ADG}}^{2}& {\sigma }_{{e}_{ADG,}{e}_{BF}}& 0& 0\\ {\sigma }_{{e}_{BF,}{e}_{ADG}}& {\sigma }_{{e}_{BF}}^{2}& 0& 0\\ 0& 0& {\sigma }_{{e}_{ADGX}}^{2}& {\sigma }_{{e}_{ADGX,}{e}_{BFX}}\\ 0& 0& {\sigma }_{{e}_{BFX,}{e}_{ADGX}}& {\sigma }_{{e}_{BFX}}^{2}\end{array}\right],$$

where ${\sigma }_{l}^{2}$ is the litter variance, ${\sigma }_{a}^{2}$ is the additive genetic variance, ${\sigma }_{e}^{2}$ is the residual variance, and other terms in the off-diagonals are the covariances between the traits (i.e., ${\sigma }_{{l}_{ADG,}{l}_{BF}}$ is the litter covariance between ADG and BF). $\mathbf{I}$ is an identity matrix and $\mathbf{H}$ is the realized relationship matrix that combines pedigree and genomic relationships in ssGBLUP. The genomic prediction was performed with both ssGBLUP and WssGBLUP using the BLUPF90 family of programs [34], which used the inverse of $\mathbf{H}$ (${\mathbf{H}}^{-1}$) as follows [24]:

$${\mathbf{H}}^{-1}= {\mathbf{A}}^{-1}+ \left[\begin{array}{cc}0& 0\\ 0& {\mathbf{G}}^{-1}- {\mathbf{A}}_{22}^{-1}\end{array}\right],$$

where ${\mathbf{G}}^{-1}$ is the inverse of the genomic relationship matrix, ${\mathbf{A}}^{-1}$ and ${\mathbf{A}}_{22}^{-1}$ are the inverses of the pedigree relationship matrices for all and genotyped individuals, respectively. The $\mathbf{G}$ matrix was created using the first method of VanRaden [16]:

$$\mathbf{G}= \frac{\mathbf{M}\mathbf{D}{\mathbf{M}}^{\mathbf{^{\prime}}}}{2\sum {\mathrm{p}}_{\mathrm{j}}(1-{\mathrm{p}}_{\mathrm{j}})},$$

where $\mathbf{M}$ is a matrix of genotypes centered for current allele frequencies, ${\mathrm{p}}_{\mathrm{j}}$ is the minor allele frequency of SNP $\mathrm{j}$, and $\mathbf{D}$ is the diagonal matrix of SNP weights. All the SNPs were presumed to have homogeneous weights in ssGBLUP, meaning that $\mathbf{D}$ is an identity matrix ($\mathbf{I}$). To ensure compatibility between $\mathbf{G}$ and ${\mathbf{A}}_{22}$ and circumvent singularity issues, $\mathbf{G}$ was tuned (scaled mean diagonal values of $\mathbf{G}$ to mean diagonal values of ${\mathbf{A}}_{22}$ and mean off-diagonals of $\mathbf{G}$ to mean off-diagonals of ${\mathbf{A}}_{22}$) and then blended with 5% of ${\mathbf{A}}_{22}$. In WssGBLUP, different weights are assigned for each SNP; therefore, $\mathbf{D}$ is no longer $\mathbf{I}$.

The algorithm for proven and young (APY) was applied to obtain ${\mathbf{G}}^{-1}$ while avoiding direct inversion of $\mathbf{G}$ [35] for lines with more than 50k genotyped animals, i.e., ML1, ML2, TL1, and TL4. Lines TL2 and TL3 used direct inversion of $\mathbf{G}$. To ensure reliable estimation of the genomic estimated breeding values (GEBV), the number of core animals corresponded to the number of eigenvalues that explained 98% of the total variation in $\mathbf{G}$ constructed using regular chip data [18]. Based on this criterion, the number of core animals randomly selected in each line was: 4200, 5400, 3400, and 5500 for ML1, ML2, TL1, and TL4, respectively.

For WssGBLUP, we calculated SNP variances from BayesR [21] and assigned those as weights for SNPs in an iterative manner. BayesR samples SNP effects from a mixture of four normal distributions with mean zero and variances equal to 0, 0.0001 $\times {\sigma }_{a}^{2}$, 0.001 $\times {\sigma }_{a}^{2}$, and 0.01 $\times {\sigma }_{a}^{2}$. Each iteration in BayesR stored individual SNP variances, and posterior SNP variance was calculated as the average variance across all the iterations. Then, the weights were re-scaled to make the trace of $\mathbf{D}$ equal to the number of SNPs. More details about BayesR weighting are described in Gualdrón-Duarte et al. [14]. This approach was exclusively implemented on the four largest lines, namely ML1, ML2, TL1, and TL4, for the growth-related traits (ADFI, ADG, BF, and LDP), using the Top40k and ChipPlusSign data to ascertain the potential benefits when the lines had a large number of genotyped pigs.

### Validation

The accuracy of genomic prediction was calculated by correlating GEBV with deregressed EBV (dEBV) for the animals in the test sets (i.e., genotyped animals), where dEBV were derived without genomic information using the method of VanRaden et al. [36]. Inflation or deflation levels were assessed as the slope (b_1_) of the regression of dEBV on GEBV. Estimates of b_1_ smaller than 1 indicated inflation of GEBV and estimates greater than 1 indicated deflation. Animals without dEBV, due to the lack of phenotypes, were removed from the test sets. Therefore, each model had different numbers of test animals. The number of test animals for each model in all lines are summarized in Additional file 1: Table S4. As only a single test set was used for each trait in each line, standard errors (SE) were computed through bootstrapping with a 1000 bootstrap replicates [37].

## Results

### Genomic prediction accuracy of maternal lines using ssGBLUP

**Fig. 1:**
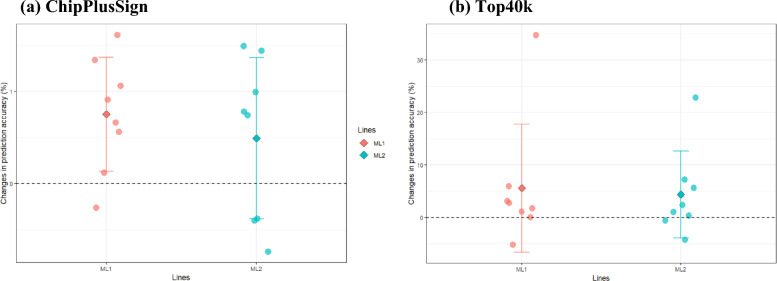
Changes in accuracy (%) for ChipPlusSign (**a**) and Top40k (**b**) compared to Chip in maternal lines. Each circle represents accuracy changes in each trait, whereas diamonds indicate the mean accuracy change across all traits in each line; error bars indicate the standard deviation across the traits

Figure 1 shows the changes in prediction accuracy (%) when using ChipPlusSign and Top40k compared to Chip for the two maternal lines. All results for prediction accuracy and changes relative to Chip (%) are summarized in Additional file 1: Tables S5 and S6, respectively. Using ChipPlusSign and Top40k led to greater accuracy than using Chip for many traits (Fig. 1). Compared to Chip, using ChipPlusSign resulted in a maximum gain of 1.6% for ADG in ML1 and of 1.5% for NSB in ML2, and in a loss of − 0.3% and − 0.7% for WWT in ML1 and ML2, respectively, and the mean accuracy across all eight traits decreased as the number of genotyped animals decreased from ML1 (76,227) to ML2 (66.608), although gain in percentage was very small (0.8 to 0.5%). When the number of animals with WGS data decreased from ML1 to ML2, using Top40k resulted in an average gain of 5.5 and 4.3% for ML1 and ML2, respectively. Using Top40k, the gain was largest for RET in ML1 (34.8%) and ML2 (22.9%), whereas the loss was largest for WWT in ML1 (− 5.2%) and ADFI in ML2 (− 4.3%). Gains in accuracy were greater with Top40k than with ChipPlusSign. Therefore, pre-selection of variants based on GWAS (ChipPlusSign and Top40k) improved prediction accuracy for most traits in maternal lines, although the gains remained small to modest.

### Genomic prediction accuracy of terminal lines using ssGBLUP

**Fig. 2:**
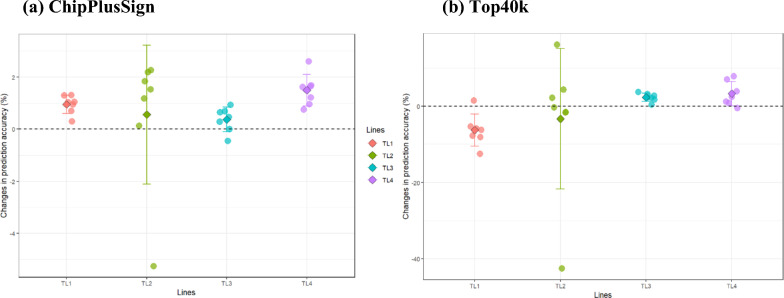
Changes in accuracy (%) for ChipPlusSign (**a**) and Top40k (**b**) compared to Chip in terminal lines. Each circle represents accuracy changes in each trait, whereas diamonds indicate the mean accuracy change across all traits in each line; error bars indicate the standard deviation across the traits

For the four terminal lines, the changes in prediction accuracy (%) of ChipPlusSign and Top40k compared to Chip are described in Fig. 2. All results for prediction accuracy and changes (%) when using Chip are summarized in Additional file 1: Tables S7 and S8. ChipPlusSign showed a consistent gain in accuracy for all traits and in all lines, except for LDPX in TL2 (− 5.3%) and for LDPX in TL3 (− 0.5%). The results obtained with ChipPlusSign showed a decreasing trend as the number of genotyped animals decreased, both in the terminal and maternal lines. In the terminal lines, the number of genotyped animals decreased from TL4 (104,644), TL1 (60,467), TL2 (41,572), to TL3 (29,328). The average gain for all seven traits was 1.0, 0.6, 0.4, and 1.5% in TL1, TL2, TL3, and TL4, respectively, and the maximum gain was 1.3% for TL1-BF, 2.3% for TL2-ADGX, 0.9% for TL3-ADFI, and 2.6% for TL4-ADGX. In contrast to results obtained with ChipPlusSign, those found with Top40k were not consistent among traits and lines. Although in TL3 and TL4, gains in accuracy were observed for most traits, except for ADFI in TL4 (− 0.5%), in TL1 and TL2, a loss of accuracy was observed for many traits (i.e., for six traits in TL1 and four traits in TL2). On average, TL3 showed the second greatest gain in accuracy for all seven traits (2.4%), with the smallest number of genotyped animals among all terminal lines. In contrast, although TL1 was the second largest genotyped line, it showed the largest loss of accuracy (− 6.3% on average). Changes in accuracy for TL1, TL2, and TL4 were − 6.3%, − 3.3%, and 3.3%, respectively, meaning that the number of genotyped animals (i.e., TL4 (104,644) > TL1 (60,467) > TL2 (41,572) > TL3 (29,328)) did not affect the gain with Top40k for terminal lines. However, the largest genotyped line (TL4) still showed the greatest average gain (3.3%). The maximum gains were 1.6% (BFX), 16.2% (ADGX), 3.7% (LDP), and 7.9% (ADGX) in TL1, TL2, TL3, and TL4, respectively. For both ChipPlusSign and Top40k, the largest standard deviation in changes of prediction accuracy (%) among all traits was observed in TL2, i.e. 2.7 and 18.4, respectively, which indicates that, in TL2, changes in accuracy depend highly on the traits. Overall, when ChipPlusSign was used, a significant but limited improvement in accuracy was found for most traits in the terminal lines (maximum 2.6% for ADGX in TL4). Results for Top40k showed a decrease in accuracy for most traits in TL1 and TL2, whereas accuracy increased for almost all traits in TL3 and TL4.

### Inflation/deflation of GEBV

**Fig. 3:**
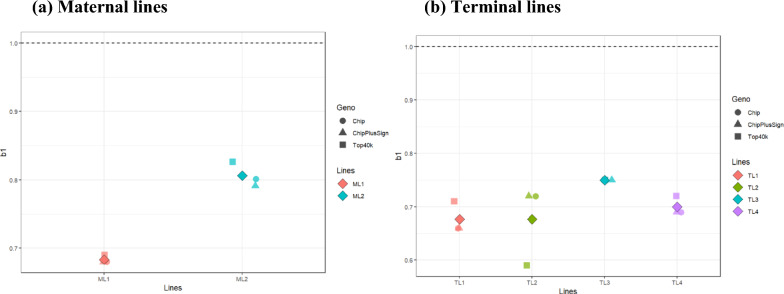
Average b_1_ values across traits for all the genotype scenarios in maternal and terminal lines**.** Diamond shape indicated the overall mean of b_1_ values for all traits and genotype panels in each line

Figure 3 shows the average estimates of b_1_ for all genotyped maternal (a) and terminal lines (b) scenarios. Estimates for each genotype scenario were averaged across all traits for each line. All other estimates for b_1_ in the maternal and terminal lines are summarized in Additional file 1: Tables S9 and S10, respectively. In the maternal lines, when the number of genotyped animals decreased (ML1 to ML2), b_1_ approached 1.0 (0.68 in ML1 and 0.81 in ML2). More specifically, all genotyping panels resulted in a smaller inflation of GEBV in ML2 than in ML1. Results for Chip, ChipPlusSign, and Top40k were similar within each line.

Compared to the results for the maternal lines, those for the terminal lines were inconsistent among traits and lines. Overall, all terminal lines showed inflated GEBV for all traits and genotyping panels. On average, the best result was obtained in TL3 (0.75), followed by TL4, TL1, and TL2 (0.70, 0.68, and 0.68, respectively). Chip, ChipPlusSign, and Top40k yielded similar results in TL3 and TL4, but in TL1 and TL2, Top40k showed either less inflation (+ 0.05) or more inflation (− 0.13), respectively, compared to Chip and ChipPlusSign.

### Genomic prediction using WssGBLUP

WssGBLUP using BayesR weights was only applied in the four largest lines (ML1, ML2, TL1, and TL4) for growth-related traits (ADFI, ADG, BF, and LDP) with Top40k and ChipPlusSign. The results for prediction accuracy and b_1_ are in Table 4 and Additional file 1: Table S11, respectively. ML1 showed no gain in accuracy when applying WssGBLUP. Using Top40k and ChipPlusSign with WssGBLUP in ML2 resulted in a 0.02 increase in accuracy in BF compared to ssGBLUP. However, a reduction of -0.09 in LDP accuracy was obtained when using the Top40k and ChipPlusSign. Results for TL1 also showed no improvement in accuracy. The greatest gains (~ 0.06) were observed in TL4 when applying WssGBLUP compared to ssGBLUP. Although there was no gain in accuracy for ADFI, for ADG, BF, and LDP gains in accuracy of 0.06, 0.03, and 0.04, respectively, were observed when WssGBLUP was used with Top40k. Similarly, ADG, BF, and LDP showed gains in accuracy of 0.03, 0.02, and 0.04, respectively, when ChipPlusSign was used with BayesR weights. Overall, WssGBLUP and regular ssGBLUP showed similar b_1_ estimates, except for a few scenarios (see Additional file 1: Table S11).

**Table 4:** Prediction accuracy of WssGBLUP compared to ssGBLUP

Line	Description	ADFI	ADG	BF	LDP
ML1	Top40k	0.37 (0.01)	0.49 (0.01)	0.51 (0.01)	0.53 (0.01)
	Top40k weighted	0.37 (0.01)	0.49 (0.01)	0.51 (0.01)	0.53 (0.01)
	ChipPlusSign	0.37 (0.01)	0.47 (0.01)	0.51 (0.01)	0.52 (0.01)
	ChipPlusSign weighted	0.37 (0.01)	0.47 (0.01)	0.51 (0.01)	0.51 (0.01)
ML2	Top40k	0.36 (0.01)	0.61 (0.01)	0.64 (0.01)	0.62 (0.01)
	Top40k weighted	0.35 (0.01)	0.62 (0.01)	0.66 (0.01)	0.53 (0.01)
	ChipPlusSign	0.37 (0.01)	0.61 (0.01)	0.63 (0.01)	0.62 (0.01)
	ChipPlusSign weighted	0.37 (0.01)	0.62 (0.01)	0.65 (0.01)	0.53 (0.01)
TL1	Top40k	0.34 (0.01)	0.45 (0.01)	0.59 (0.01)	0.56 (0.01)
	Top40k weighted	0.34 (0.01)	0.45 (0.01)	0.59 (0.01)	0.55 (0.01)
	ChipPlusSign	0.36 0.01)	0.49 (0.01)	0.61 (0.02)	0.60 (0.01)
	ChipPlusSign weighted	0.36 (0.01)	0.50 (0.01)	0.60 (0.02)	0.60 (0.01)
TL4	Top40k	0.39 (0.01)	0.51 (0.01)	0.60 (0.01)	0.59 (0.01)
	Top40k weighted	0.39 (0.01)	0.57 (0.01)	0.63 (0.01)	0.63 (0.01)
	ChipPlusSign	0.40 (0.01)	0.51 (0.01)	0.60 (0.01)	0.57 (0.01)
	ChipPlusSign weighted	0.40 (0.01)	0.54 (0.01)	0.62 (0.01)	0.61 (0.01)

ML1: maternal line 1; ML2: maternal line 2; TL1: terminal line 1; TL4: terminal line4; ADFI: average daily feed intake; ADG: average daily gain; BF: backfat thickness; LDP: loin depth; Top40k: top40k preselected genotype panel; Top40k weighted: top40k using BayesR weighting; ChipPlusSign: ChipPlusSign preselected genotype panel; ChipPlusSign weighted: ChipPlusSign using BayesR weighting

Standard errors are in parenthesis

## Discussion

The current study investigated the impact of using large-scale WGS data for genomic prediction through ssGBLUP and WssGBLUP in maternal and terminal pig lines. This is the first study that applies ssGBLUP to large-scale WGS pig datasets, with up to 1.8k sequenced and 104k imputed sequenced animals per line. We used two sets of preselected WGS variants to compare the performance of genomic predictions to that of the regular SNP chip. Our results show that preselected variants can outperform the regular SNP chip in genomic prediction. However, this advantage is not consistent across the lines and traits examined, and the improvement achieved was relatively limited, as previously reported for the same data using a BayesR approach [8]. In addition, we observed that WssGBLUP using posterior variances from the BayesR as SNP weights has the potential to improve prediction accuracy, especially for the largest genotyped populations. In this Discussion section, we address three points: (1) the impact of the method used for preselecting WGS variants on genomic prediction, (2) the use of WGS data for genomic prediction in pigs, and (3) the comparison of weighted with non-weighted ssGBLUP.

### Impact of the method used for preselecting WGS variants on genomic prediction

Theoretically, using WGS data can improve genomic predictions because they cover the entire genome, and thus it is assumed that they include the causative variants. As a result, genomic prediction using WGS data does not rely on the LD between SNPs and causative variants but can directly use the causative variants [38]. Thus, using WGS data in genomic prediction is expected to increase accuracy because the variants present in the data can explain a larger proportion of the genetic variance than the SNPs on the genotyping chip. However, several studies have reported that using all variants included in WGS data did not improve prediction accuracies [6, 7, 10]. A plausible reason is that WGS data include many redundant SNPs. Since WGS data have millions of SNPs across the entire genome, neighboring SNPs are probably in strong LD with causative variants or with other SNPs located in specific genomic blocks. This suggests that many SNPs are correlated and provide redundant information. Therefore, fitting all SNPs from WGS data into the prediction model could lead to biased GEBV (i.e., dispersion bias measured by the slope of the regression of dEBV on GEBV). To avoid bias, many studies have investigated the potential benefit of preselecting variants that have been shown to be significantly associated with the trait for genomic prediction [2–4]. Thus, in the current study, two different preselected genotype panels, ChipPlusSign and Top40k, were designed and compared to the regular chip data for genomic prediction. These panels were constructed following different assumptions, as in Ros-Freixedes et al. [8]. For ChipPlusSign, significant variants (p ≤ 10^–6^) based on GWAS were added to the regular SNP chip with an expectation of better prediction accuracy if the significant SNPs had large effects or were causative and were not present on the regular SNP chip. Incorporating preselected, significant SNPs into the regular SNP chip has been investigated in many studies with WGS data [3, 13, 39]. Top40k was created to mimic the number of SNPs in the regular medium-density SNP chips used for routine genomic evaluation in many livestock (e.g., pigs, cattle, and chickens). Similar to most regular SNP chips that contain evenly spaced SNPs, Top40k also consisted of 40k SNPs from WGS that had the lowest p-value (i.e., from GWAS) in each of 40k consecutive non-overlapping 55-kb window. Therefore, we expected gains in prediction accuracy if those preselected 40k SNPs from WGS data were more informative and explained a larger proportion of genetic variation than the SNPs on the regular chip. In this study, the Top40k sets in the multi-trait models (ADFI, GROWTH, and LOIN) were combined, generating about 80k SNPs (see Additional file 1: Tables S2 and S3), which differs from the study of Ros-Freixedes et al. [8], who used the same datasets but only with single-trait models.

Among the preselected genotyping sets, ChipPlusSign showed small to moderate gains in accuracy for many traits in the maternal and terminal lines. This panel also showed the most consistent results across lines and traits, with gains in accuracy observed in most cases, but within a limited range (from 0.1 to 2.6%). ChipPlusSign also showed greater robustness in the performance of genomic prediction than Top40k when the genomic prediction was performed using BayesR on the same data [8].

Several studies have been conducted to investigate genomic prediction by adding preselected variants to the regular chip data using real or simulated datasets [2, 3, 17, 39]. In US Holstein cattle, VanRaden et al. [3] investigated the reliability of GEBV for 33 traits when preselected SNPs (N = 16k) from WGS data were added to a 60k SNP chip. They reported an increase in reliability (= squared accuracy) of up to a 4.8 percentage point (15.35%), with an average increase of 2.7 points (9.15%) compared to the reliability obtained with the 60k SNP chip. However, when Fragomeni et al. [2] investigated the performance of ssGBLUP using the same preselected variants set as used by VanRaden et al. [3], almost no gain in reliability (0.92%) was observed, although reliabilities were greater than obtained by VanRaden et al. [3]. One major difference between these two studies was the multistep method used for genomic prediction by VanRaden et al. [3] and ssGBLUP by Fragomeni et al. [2]. With ssGBLUP, all information from genotyped and non-genotyped animals was combined, which represented a massive amount of data. In such a scenario, gains in reliability are less likely if the selected variants are redundant, not truly causative, or have a small effect on the trait of interest. Our results agree with those of Fragomeni et al. [2], especially our results obtained with ChipPlusSign.

In a simulation study, Jang et al. [17] investigated the dimensionality of genomic information for variant selection and genomic prediction with WGS data. Their results showed that for populations with a small *Ne* the maximum gain in accuracy ranged from 0.86 to 1.98% when either significant variants or hundreds of variants with a large effect size that were preselected from GWAS were added to a 50k SNP chip. In their scenario, they simulated a *Ne* of 20, which is close to the *Ne* in pig populations (32–48) [19] although the *Ne* of real pig data seemed to be greater than 20 due to the variation between the populations.

In our study, the results obtained with Top40k depended highly on the trait and line. Top40k showed the greatest gain in accuracy for RET across all maternal lines (22.9 to 34.8%) but relatively marginal gains or losses for the other traits. In the terminal lines, the results for Top40k fluctuated more among lines, with increases or decreases in accuracy. The large improvement observed for reproduction or fertility traits in the maternal lines might be due to the nature of these traits and the lack of informative SNPs for these traits on the regular SNP chip. For example, the lack of informative SNPs for fertility traits led to a recent change in the SNP chip for beef cattle evaluations (https://www.angus.org/AGI/global/AngusGS.pdf). In addition, heritabilities for RET were lower than for other traits. Consequently, this trait had the lowest prediction accuracies (0.14 in ML1 and 0.20 in ML2 with Chip) among the other traits in the maternal lines. Thus, genomic prediction using preselected genotype data is expected to lead to greater improvements in accuracy if the SNPs on the regular chip do not explain a large proportion of the genetic variance. Therefore, we speculated that there would be more informative SNPs in Top40k for RET, which are not included in the regular SNP chip. Likewise, the small differences in accuracy observed between Chip and Top40k were likely to result from variants on these chips capturing similar proportions of genetic variance and having similar LD patterns across the genome. Although we observed inconsistent results between the terminal lines using Top40k, ADGX showed the highest gain (16.2% in TL2), which was recorded in the crossbred animals. ADGX was investigated in the GROWTH model along with three correlated traits (ADG, BF, and BFX), and Top40k was created based on GWAS for ADG and BF, individually, after which each Top40k was combined for genomic prediction. Thus, this result shows the potential benefits for improving genomic prediction of traits recorded in crossbred animals if many phenotypes are available for both purebreds and crossbreds. However, crossbred traits were not directly used for preselecting variants because WGS information was available only on purebred animals.

The marginal gains in accuracy that were observed for most traits when using ChipPlusSign and Top40k raised a question about the amount of information that has been used for preselecting the variants and performing genomic predictions. Examining the dimensionality of the genomic information can help assess the number of genotyped animals needed to maximize the percentage of variant discoveries in GWAS and gains in prediction accuracy [17]. According to Jang et al. [17], using a number of genotyped animals that is equal to the number of eigenvalues that explain 98% of the variance of $\mathbf{G}$ is sufficient to capture the most informative variants in the populations with a large effective size (*Ne* = 200). However, only a small proportion of the causative variants were discovered for highly polygenic traits and their study showed that populations with a smaller effective size (*Ne* = 20) required much more data to capture the causative variants. For example, when 30k genotyped animals were used in GWAS for highly polygenic traits, only three causative variants that explained 3.9% of the genetic variance were identified. In addition, adding preselected variants to regular chip data yielded a maximum gain in accuracy of nearly 2% for the scenarios with *Ne* = 20. In the current study, the number of WGS animals used for GWAS ranged from 29 to 104k, which is the largest WGS data used for GWAS in pigs by far. However, fine-mapping the causative variants was still challenging and the benefits for genomic predictions were limited [8]. Since commercial pig breeding populations have small *Ne* and most of the traits are highly polygenic, to capture the most informative variants, it is necessary to have a very large number of WGS animals (i.e., 30k) with many progeny records [17].

In an initial series of analyses (results not shown), we used only significant variants (TopSign) for genomic prediction, and found no benefit compared to Chip. In fact, we observed a loss in accuracy for most traits and lines. Depending on the line and trait, the number of variants in TopSign ranged from 6 to 1705. Fragomeni et al. [13] reported that the maximum accuracy of genomic predictions could be obtained when the true causative variants were identified with their exact substitution effects (i.e., true effects of variants), position in the genome, and the proportion of genetic variance explained by each variant. Therefore, our results revealed that the variants in TopSign might not be truly causative, resulting in the use of these variants to underperform the regular SNP chip. Based on this, the small gains in accuracy observed with ChipPlusSign and Top40k could be due to an increase in the number of SNPs in these panels (e.g., the number of SNPs on Chip, ChipPlusSign, and Top40k in ML1 was 40,592, 41,364, and 80,308) rather than to the significance of the preselected SNPs. In a simulation study that mimicked a population with a small *Ne*, Jang et al. [17] demonstrated that adding a few hundred to thousands of SNPs to the regular chip data resulted only in marginal improvements in prediction accuracy, although they explained a significant proportion of the genetic variance.

### Using WGS data for genomic prediction in pigs

With the decrease in sequencing costs, the use of WGS data for genomic prediction in livestock (e.g., sheep, beef cattle, dairy cattle, and pigs) is more affordable than in the past. Several studies have reported marginal or no benefits of using WGS for genomic prediction in sheep, beef, and dairy cattle [2, 3, 11, 12, 39]. Compared to other livestock species, in pigs, there is a scarcity of studies focused on the use of WGS data for genomic prediction. Moreover, those studies have primarily used smaller datasets [6, 7], with the number of imputed sequenced pigs being less than 7k. As the number of variants in WGS increases, more samples are required to resolve the well-known issue of ‘$N << p$’, where $N$ is the sample size, and $p$ is the number of variants. If sample size is not large enough, estimating SNP effects and identifying causative SNPs could be difficult, especially for the populations with small *Ne* and highly polygenic traits.

In the current study, the number of sequenced animals ranged from ~ 380 to 1.8k across lines, which represented nearly 2% of the population in each line [8]. However, depending on the line, the WGS information was imputed for 29k to 104k animals. Applying large-scale WGS data to preselect variants through GWAS and using those variants for genomic prediction showed limited improvement in our study and in Ros-Freixedes et al. [8], similar to previous studies that used a small number of animals with WGS [6, 7]. Increasing the sample size could enhance the power to detect causative variants and improve genomic predictions [38, 40]. However, commercial pig breeding populations are highly structured and have a small *Ne*. Therefore, increasing only the sample size might not help improve the performance of both variant selection and genomic prediction. Jang et al. [17] reported that using animals with greater EBV accuracy (i.e., with more progeny phenotypes) helped to better identify the causative variants compared to using animals that had lower EBV accuracy. Therefore, selecting high-accuracy animals and using them as a variant discovery set could be a possible strategy. In addition, a smaller *Ne* implies a lower *Me*, which means that the number of SNPs on the 50k chip is likely sufficient to represent the chromosome segments in the populations to cover the entire genome. Theoretically, the ideal number of SNPs on chip panels can be calculated as 12 $\times$ *Me* [41]. In commercial pig breeding populations, *Me* ranges from ~ 4k to 6k [19], meaning that the number of SNPs genotyped should range from 48 to 72k; therefore, a 50k chip may be sufficient. This could explain why limited gains are obtained when using WGS data in pigs. Therefore, the ultimate goal of using WGS data for genomic prediction is to design a customized SNP chip that consists of the already-known SNPs in commercial chip data and those discovered as significant for economically important traits. Here, transcriptomic and functional information could help identify important, non-redundant SNPs [42].

Another possible reason for the limited benefit of using WGS data could be the imputation accuracy [20, 43]. The ideal situation to use WGS data is to sequence all the animals in the population without imputation from low-density genotype to the sequence level. However, as sequencing the entire population is still not feasible, imputation is inevitable when dealing with WGS data. Because the reference data for imputation consisted of a limited number of sequenced animals, sequencing more animals and using more robust statistical tools to impute alleles accurately are required.

### Comparison of weighted and non-weighted ssGBLUP

The current study performed genomic prediction using ssGBLUP for the following reasons: it can consider multi-trait models and virtually any mixed linear model used for genomic evaluation (e.g., maternal effects) [23]. The study on the same data used here by Ros-Freixedes [8] applied a simplified single-trait model through BayesR. Furthermore, ssGBLUP is a widely recognized method for routine genomic evaluation in various livestock species. This is due to its ability to use all available data from genotyped and non-genotyped individuals [23, 27–29]. A major assumption of GBLUP-based methods is that the markers have homogeneous variance, which is a reasonable approximation for most livestock traits due to their highly polygenic nature [24, 27]. However, biologically, this assumption does not hold because not all markers in the genome explain the same proportion of variance [44]. Therefore, assigning heterogeneous variance per marker for genomic prediction has been investigated in several studies [14, 45, 46]. Weighting SNPs in ssGBLUP was initially proposed by Wang et al. [46] by assigning unequal SNP variances based on the square of the SNP effect estimates weighted by their allele frequencies. However, this method resulted in reductions in accuracy of genomic predictions and extra biases over iterations due to the extreme values that were obtained for SNP variances, especially for polygenic traits [47, 48].

Following the increased accuracy reported by Gualdrón-Duarte et al. [14], we used the posterior SNP variances from BayesR as SNP weights. In BayesR, SNP effects are sampled from a mixture of four normal distributions with mean zero and variances equivalent to the following classes: 0, 0.0001 ${\sigma }_{g}^{2}$, 0.001 ${\sigma }_{g}^{2}$, and 0.01 ${\sigma }_{g}^{2}$ [22], which we expected to lead to a better weighting matrix, i.e., closer to weighting based on the true variance of each SNP. Our results showed that WssGBLUP outperformed ssGBLUP for ADG, BF, and LDP in TL4 for both Top40k and ChipPlusSign by up to 0.06 in prediction accuracy, although the gains were still marginal. However, the results of WssGBLUP for the other traits in ML1, ML2, and TL1 were similar to those of ssGBLUP. We expected almost no gain with WssGBLUP, especially for the largest genotyped population (TL4), since the SNP effects were likely dominated by a large amount of data in the single-step system, meaning that the impact of the prior is less critical. However, we observed potential room for improvement in predictions when using the posterior variance of BayesR, even with a large amount of data. In other words, although the volume of data could overwhelm the a priori assumption for SNP effects, we can still observe the benefits if the variances used as SNP weights are sufficiently accurate.

## Conclusions

Preselection of significant variants from WGS data and their use in genomic prediction can help to improve genomic predictions in maternal and terminal pig lines with tens of thousands of sequenced/imputed animals. However, limited gains are noted even in large populations. Improvements may be observed when significant variants for some traits are not already represented by the SNPs present on the commercial SNP chips and with that, traits that have a limited accuracy may experience extra gains. Weighting SNPs using BayesR variances slightly improved prediction accuracies. The performance of genomic predictions using preselected variant sets depends highly on the population structure, number of genotyped animals, and method used to select the variants.

## Supplementary Information

**Additional file 1: Table S1.** Combination of selected variants for each model. ADFI: average daily feed intake; ADG: average daily gain; BF: backfat thickness; LDP: loin depth; TNB: total number of piglets born; NSB: number of stillborn; RET: return to oestrus seven days after weaning; WWT: litter weaning weight; Top40k: Top40k preselected genotype panel; ChipPlusSign: ChipPlusSign preselected genotype panel. **Table S2.** Number of animals and SNPs for the pre-selected SNP panels in the maternal lines. ML1: maternal line 1; ML2: maternal line 2; Top40k: Top40k preselected genotype panel; *ChipPlusSign: ChipPlusSign preselected genotype panel. **Table S3.** Number of animals and SNPs for the pre-selected SNP panels in the terminal lines. TL1: terminal line 1; TL2: terminal line 2; TL3: terminal line 3; TL4: terminal line 4; Top40k: Top40k preselected genotype panel; ChipPlusSign: ChipPlusSign preselected genotype panel. **Table S4.** Number of training and test animals for each model in the two maternal and four terminal lines. ADFI: average daily feed intake; ADG: average daily gain; BF: backfat thickness; LDP: loin depth; TNB: total number of piglets born; NSB: number of stillborn; RET: return to oestrus seven days after weaning; WWT: litter weaning weight; ADGX: ADG recorded in crossbred; BFX: BF recorded in crossbred; LDPX: LDP recorded in crossbred. **Table S5.** Prediction accuracy of the maternal lines using ssGBLUP. ML1: maternal line 1; ML2: maternal line 2; ADFI: average daily feed intake; ADG: average daily gain; BF: backfat thickness; LDP: loin depth; TNB: total number of piglets born; NSB: number of stillborn; RET: return to oestrus seven days after weaning; WWT: litter weaning weight; standard errors are presented in parenthesis. **Table S6.** Accuracy gain and reduction (%) compared to Chip data in the maternal lines. ML1: maternal line 1; ML2: maternal line 2; ADFI: average daily feed intake; ADG: average daily gain; BF: backfat thickness; LDP: loin depth; TNB: total number of piglets born; NSB: number of stillborn; RET: return to oestrus seven days after weaning; WWT: litter weaning weight. **Table S7.** Prediction accuracy of the terminal lines using ssGBLUP. TL1: terminal line 1; TL2: terminal line 2; TL3: terminal line 3; TL4: terminal line 4; ADFI: average daily feed intake; ADG: average daily gain; BF: backfat thickness; LDP: loin depth; ADGX: ADG recorded in crossbred; BFX: BF recorded in crossbred; LDPX: LDP recorded in crossbred; standard errors are presented in parenthesis. **Table S8.** Accuracy gain and reduction (%) compared to Chip data in the terminal lines. TL1: terminal line 1; TL2: terminal line 2; TL3: terminal line 3; TL4: terminal line 4; ADFI: average daily feed intake; ADG: average daily gain; BF: backfat thickness; LDP: loin depth; ADGX: ADG recorded in crossbred; BFX: BF recorded in crossbred; LDPX: LDP recorded in crossbred. **Table S9.** Slope (b_1_) of the regression of dEBV on GEBV for the maternal lines using ssGBLUP. ML1: maternal line 1; ML2: maternal line 2; ADFI: average daily feed intake; ADG: average daily gain; BF: backfat thickness; LDP: loin depth; TNB: total number of piglets born; NSB: number of stillborn; RET: return to oestrus seven days after weaning; WWT: litter weaning weight; Standard errors are presented in parenthesis. **Table S10.** Slope (b_1_) of the regression of dEBV on GEBV for the terminal lines using ssGBLUP. TL1: terminal line 1; TL2: terminal line 2; TL3: terminal line 3; TL4: terminal line 4; ADFI: average daily feed intake; ADG: average daily gain; BF: backfat thickness; LDP: loin depth; ADGX: ADG recorded in crossbred; BFX: BF recorded in crossbred; LDPX: LDP recorded in crossbred; Standard errors are presented in parenthesis. **Table S11.** Slope (b_1_) of the regression of dEBV on GEBV for WssGBLUP compared to ssGBLUP. ML1: maternal line 1; ML2: maternal line 2; TL1: terminal line 1; TL4: terminal line 4; ADFI: average daily feed intake; ADG: average daily gain; BF: backfat thickness; LDP: loin depth; Top40k: top40k preselected genotype panel; Top40k weighted: top40k using BayesR weighting; ChipPlusSign: ChipPlusSign preselected genotype panel; ChipPlusSign weighted: ChipPlusSign using BayesR weighting; Standard errors are presented in parenthesis

## Data Availability

The datasets generated and analyzed in this study are from the PIC breeding programme and not publicly available.
