# Supplementary material for: Using pre-selected variants from large-scale whole-genome sequence data for single-step genomic predictions in pigs
Source: Genet Sel Evol. 2023 Jul 26;55:55. doi: 10.1186/s12711-023-00831-0 (PMC10373252; doi:10.1186/s12711-023-00831-0)
Supplement: Supplementary file 1 — Additional file 1: Table S1. Combination of selected variants for each model. ADFI: average daily feed intake; ADG: average daily gain; BF: backfat thickness; LDP: loin depth; TNB: total number of piglets born; NSB: number of stillborn; RET: return to oestrus seven days after weaning; WWT: litter weaning weight; Top40k: Top40k preselected genotype panel; ChipPlusSign: ChipPlusSign preselected genotype panel. Table S2. Number of animals and SNPs for the pre-selected SNP panels in the maternal lines. ML1: maternal line 1; ML2: maternal line 2; Top40k: Top40k preselected genotype panel; *ChipPlusSign: ChipPlusSign preselected genotype panel. Table S3. Number of animals and SNPs for the pre-selected SNP panels in the terminal lines. TL1: terminal line 1; TL2: terminal line 2; TL3: terminal line 3; TL4: terminal line 4; Top40k: Top40k preselected genotype panel; ChipPlusSign: ChipPlusSign preselected genotype panel. Table S4. Number of training and test animals for each model in the two maternal and four terminal lines. ADFI: average daily feed intake; ADG: average daily gain; BF: backfat thickness; LDP: loin depth; TNB: total number of piglets born; NSB: number of stillborn; RET: return to oestrus seven days after weaning; WWT: litter weaning weight; ADGX: ADG recorded in crossbred; BFX: BF recorded in crossbred; LDPX: LDP recorded in crossbred. Table S5. Prediction accuracy of the maternal lines using ssGBLUP. ML1: maternal line 1; ML2: maternal line 2; ADFI: average daily feed intake; ADG: average daily gain; BF: backfat thickness; LDP: loin depth; TNB: total number of piglets born; NSB: number of stillborn; RET: return to oestrus seven days after weaning; WWT: litter weaning weight; standard errors are presented in parenthesis. Table S6. Accuracy gain and reduction (%) compared to Chip data in the maternal lines. ML1: maternal line 1; ML2: maternal line 2; ADFI: average daily feed intake; ADG: average daily gain; BF: backfat thickness; LDP: loin depth; TNB: total num [file 12711_2023_831_MOESM1_ESM.docx]

**Supplementary table 1. Combination of selected variants for each model**

| **Model** | **SNP panel** | **Maternal lines** | **Terminal lines** |
| --- | --- | --- | --- |
| ADFI | Top40k, ChipPlusSign | ADG + ADFI | ADG + ADFI |
| GROWTH | Top40k, ChipPlusSign | ADG + BFP | ADG + BFP |
| LOINDEPT | Top40k, ChipPlusSign | ADG + LDP | ADG + LDP |
| REPROD | Top40k, ChipPlusSign | TNB + NSB | - |
| RET | Top40k, ChipPlusSign | RET | - |
| WWT | Top40k, ChipPlusSign | WWT | - |

**Supplementary table 2. Number of animals and SNPs for pre-selected SNP panels in maternal lines**

| **Model** | **SNP panel** | **ML1** | | **ML2** | |
| --- | --- | --- | --- | --- | --- |
|  |  | **SNPs** | **Animals** | **SNPs** | **Animals** |
| ADFI | ChipPlusSign | 41,364 | 76,227 | 43,325 | 66,608 |
|  | Top40k | 80,308 | 76,244 | 80,819 | 66,608 |
| GROWTH | ChipPlusSign | 41,909 | 76,227 | 43,819 | 66,608 |
|  | Top40k | 80,613 | 76,230 | 79,984 | 66,608 |
| LOIN | ChipPlusSign | 41,742 | 76,227 | 43,618 | 66,608 |
|  | Top40k | 79,605 | 76,230 | 79,625 | 66,608 |
| REPROD | ChipPlusSign | 40,707 | 76,227 | 42,907 | 66,608 |
|  | Top40k | 41,180 | 76,214 | 41,173 | 66,561 |
| RET | ChipPlusSign | 40,624 | 76,227 | 42,819 | 66,608 |
|  | Top40k | 41,171 | 76,177 | 41,168 | 66,300 |
| WWT | ChipPlusSign | 40,772 | 76,227 | 42,777 | 66,608 |
|  | Top40k | 41,173 | 76,244 | 41,165 | 66,608 |

**Supplementary table 3. Number of animals and SNPs for pre-selected SNP panels in terminal lines**

| **Model** | **SNP panel** | **TL1** | | **TL2** | | **TL3** | | **TL4** | |
| --- | --- | --- | --- | --- | --- | --- | --- | --- | --- |
|  |  | **SNPs** | **Animals** | **SNPs** | **Animals** | **SNPs** | **Animals** | **SNPs** | **Animals** |
| ADFI | ChipPlusSign | 36,305 | 60,467 | 40,744 | 41,572 | 40,267 | 29,328 | 43,814 | 104,644 |
|  | Top40k | 79,594 | 59,453 | 78,714 | 41,507 | 80,070 | 29,195 | 80,153 | 104,659 |
| GROWTH | ChipPlusSign | 36,792 | 60,467 | 41,578 | 41,572 | 40,432 | 29,328 | 44,734 | 104,645 |
|  | Top40k | 79,078 | 59,593 | 79,112 | 41,533 | 80,155 | 29,307 | 80,899 | 104,659 |
| LOIN | ChipPlusSign | 36,796 | 60,467 | 40,798 | 41,572 | 40,429 | 29,328 | 44,305 | 104,645 |
|  | Top40k | 78,789 | 59,615 | 77,878 | 41,535 | 80,535 | 29,286 | 81,439 | 104,659 |

**Supplementary table 4. Number of test animals for each trait in all lines**

| **Line** | **ADFI** | **ADG** | **BF** | **LDP** | **TNB** | **NSB** | **RET** | **WWT** |
| --- | --- | --- | --- | --- | --- | --- | --- | --- |
| ML1 | 8,387 | 10,614 | 8,418 | 8,422 | 425 | 362 | 246 | 332 |
| ML2 | 6,976 | 9,237 | 9,237 | 7,363 | 399 | 401 | 220 | 282 |
| **Line** | **ADFI** | **ADG** | **BF** | **ADGX** | **BFX** | **LDP** | | **LDPX** |
| TL1 | 5,970 | 5,970 | 5,970 | 5,970 | 5,970 | 5,965 | | 5,943 |
| TL2 | 3,720 | 3,720 | 3,720 | 3,720 | 3,720 | 3,720 | | 2,858 |
| TL3 | 1,808 | 2,324 | 2,324 | 2,324 | 2,324 | 2,324 | | 2,324 |
| TL4 | 9,434 | 11,308 | 11,308 | 11,308 | 11,308 | 11,308 | | 11,308 |

**Supplementary table 5.** **Prediction accuracy of maternal lines using ssGBLUP**

| **Line** | **SNP panel** | **ADFI** | **ADG** | **BF** | **LDP** | **TNB** | **NSB** | **RET** | **WWT** |
| --- | --- | --- | --- | --- | --- | --- | --- | --- | --- |
| ML1 | Chip | 0.361 | 0.460 | 0.509 | 0.515 | 0.406 | 0.358 | 0.139 | 0.304 |
|  | ChipPlusSign | 0.366 | 0.468 | 0.514 | 0.519 | 0.410 | 0.360 | 0.139 | 0.303 |
|  | Top40k | 0.368 | 0.488 | 0.515 | 0.530 | 0.419 | 0.358 | 0.187 | 0.288 |
| ML2 | Chip | 0.371 | 0.611 | 0.629 | 0.611 | 0.348 | 0.366 | 0.204 | 0.284 |
|  | ChipPlusSign | 0.374 | 0.609 | 0.634 | 0.620 | 0.351 | 0.371 | 0.204 | 0.282 |
|  | Top40k | 0.355 | 0.608 | 0.644 | 0.618 | 0.368 | 0.392 | 0.251 | 0.285 |

**Supplementary table 6. Accuracy gain and reduction (%) compared to Chip data in maternal lines**

| **Line** | **SNP panel** | **ADFI** | **ADG** | **BF** | **LDP** | **TNB** | **NSB** | **RET** | **WWT** | **Mean** |
| --- | --- | --- | --- | --- | --- | --- | --- | --- | --- | --- |
| ML1 | ChipPlusSign | 1.3 | 1.6 | 0.9 | 0.7 | 1.1 | 0.6 | 0.1 | -0.3 | 0.8 |
|  | Top40k | 1.7 | 5.9 | 1.1 | 2.8 | 3.1 | 0.0 | 34.8 | -5.2 | 5.5 |
| ML2 | ChipPlusSign | 0.8 | -0.4 | 0.7 | 1.4 | 1.0 | 1.5 | -0.4 | -0.7 | 0.5 |
|  | Top40k | -4.3 | -0.6 | 2.4 | 1.1 | 5.6 | 7.2 | 22.9 | 0.3 | 4.3 |

**Supplementary table 7. Prediction accuracy of terminal lines using ssGBLUP**

| **Line** | **SNP panel** | **ADFI** | **ADG** | **BF** | **ADGX** | **BFX** | **LDP** | **LDPX** |
| --- | --- | --- | --- | --- | --- | --- | --- | --- |
| TL1 | Chip | 0.359 | 0.488 | 0.600 | 0.334 | 0.616 | 0.596 | 0.321 |
|  | ChipPlusSign | 0.363 | 0.494 | 0.608 | 0.337 | 0.618 | 0.600 | 0.324 |
|  | Top40k | 0.338 | 0.427 | 0.563 | 0.308 | 0.625 | 0.548 | 0.304 |
| TL2 | Chip | 0.295 | 0.483 | 0.600 | 0.178 | 0.481 | 0.473 | 0.050 |
|  | ChipPlusSign | 0.301 | 0.492 | 0.607 | 0.182 | 0.489 | 0.474 | 0.047 |
|  | Top40k | 0.301 | 0.504 | 0.598 | 0.207 | 0.474 | 0.465 | 0.029 |
| TL3 | Chip | 0.356 | 0.629 | 0.546 | 0.392 | 0.414 | 0.611 | 0.572 |
|  | ChipPlusSign | 0.360 | 0.632 | 0.547 | 0.394 | 0.416 | 0.611 | 0.570 |
|  | Top40k | 0.366 | 0.640 | 0.548 | 0.401 | 0.427 | 0.634 | 0.585 |
| TL4 | Chip | 0.397 | 0.497 | 0.594 | 0.262 | 0.587 | 0.564 | 0.483 |
|  | ChipPlusSign | 0.403 | 0.505 | 0.599 | 0.269 | 0.592 | 0.571 | 0.491 |
|  | Top40k | 0.395 | 0.510 | 0.601 | 0.283 | 0.593 | 0.587 | 0.517 |

**Supplementary table 8. Accuracy gain and reduction (%) compared to Chip data in terminal lines**

| **Line** | **SNP panel** | **ADFI** | **ADG** | **BF** | **ADGX** | **BFX** | **LDP** | **LDPX** | **Mean** |
| --- | --- | --- | --- | --- | --- | --- | --- | --- | --- |
| TL1 | ChipPlusSign | 1.0 | 1.3 | 1.3 | 1.0 | 0.3 | 0.7 | 1.1 | 1.0 |
|  | Top40k | -5.8 | -12.5 | -6.2 | -7.7 | 1.6 | -8.1 | -5.3 | -6.3 |
| TL2 | ChipPlusSign | 2.2 | 1.9 | 1.2 | 2.3 | 1.5 | 0.1 | -5.3 | 0.6 |
|  | Top40k | 2.2 | 4.4 | -0.3 | 16.2 | -1.5 | -1.6 | -42.6 | -3.3 |
| TL3 | ChipPlusSign | 0.9 | 0.5 | 0.3 | 0.7 | 0.7 | 0.0 | -0.5 | 0.4 |
|  | Top40k | 2.7 | 1.7 | 0.4 | 2.4 | 3.2 | 3.7 | 2.2 | 2.4 |
| TL4 | ChipPlusSign | 1.7 | 1.7 | 1.0 | 2.6 | 0.8 | 1.2 | 1.6 | 1.5 |
|  | Top40k | -0.5 | 2.5 | 1.2 | 7.9 | 0.9 | 4.0 | 7.1 | 3.3 |

**Supplementary table 9. b_1_ of maternal lines using ssGBLUP**

| **Line** | **SNP panel** | **ADFI** | **ADG** | **BF** | **LDP** | **TNB** | **NSB** | **RET** | **WWT** | **Mean** |
| --- | --- | --- | --- | --- | --- | --- | --- | --- | --- | --- |
| ML1 | Chip | 0.91 | 0.56 | 0.61 | 0.66 | 0.80 | 0.75 | 0.47 | 0.71 | 0.68 |
|  | ChipPlusSign | 0.90 | 0.56 | 0.61 | 0.66 | 0.80 | 0.76 | 0.47 | 0.70 | 0.68 |
|  | Top40k | 0.89 | 0.60 | 0.64 | 0.68 | 0.72 | 0.85 | 0.47 | 0.65 | 0.69 |
| ML2 | Chip | 0.99 | 0.77 | 0.67 | 0.79 | 0.77 | 1.06 | 0.56 | 0.80 | 0.80 |
|  | ChipPlusSign | 0.98 | 0.74 | 0.64 | 0.77 | 0.78 | 1.07 | 0.56 | 0.79 | 0.79 |
|  | Top40k | 0.90 | 0.73 | 0.69 | 0.77 | 0.89 | 1.23 | 0.68 | 0.72 | 0.83 |

**Supplementary table 10. b_1_ of terminal lines using ssGBLUP**

| **Line** | **SNP panel** | **ADFI** | **ADG** | **BF** | **ADGX** | **BFX** | **LDP** | **LDPX** | **Mean** |
| --- | --- | --- | --- | --- | --- | --- | --- | --- | --- |
| TL1 | Chip | 0.74 | 0.62 | 0.70 | 0.76 | 0.58 | 0.69 | 0.50 | 0.66 |
|  | ChipPlusSign | 0.74 | 0.63 | 0.70 | 0.76 | 0.59 | 0.70 | 0.51 | 0.66 |
|  | Top40k | 0.68 | 0.64 | 0.72 | 0.77 | 0.79 | 0.69 | 0.67 | 0.71 |
| TL2 | Chip | 0.63 | 0.64 | 0.58 | 0.79 | 0.60 | 0.60 | 1.23 | 0.72 |
|  | ChipPlusSign | 0.64 | 0.64 | 0.57 | 0.80 | 0.60 | 0.60 | 1.16 | 0.72 |
|  | Top40k | 0.57 | 0.59 | 0.57 | 0.85 | 0.58 | 0.55 | 0.43 | 0.59 |
| TL3 | Chip | 1.08 | 0.68 | 0.63 | 0.91 | 0.68 | 0.60 | 0.66 | 0.75 |
|  | ChipPlusSign | 1.08 | 0.68 | 0.63 | 0.91 | 0.68 | 0.59 | 0.69 | 0.75 |
|  | Top40k | 1.07 | 0.66 | 0.62 | 0.95 | 0.73 | 0.55 | 0.65 | 0.75 |
| TL4 | Chip | 0.86 | 0.65 | 0.65 | 0.62 | 0.68 | 0.71 | 0.66 | 0.69 |
|  | ChipPlusSign | 0.85 | 0.65 | 0.64 | 0.63 | 0.67 | 0.71 | 0.67 | 0.69 |
|  | Top40k | 0.80 | 0.66 | 0.66 | 0.75 | 0.72 | 0.71 | 0.74 | 0.72 |

**Supplementary table 11. b_1_ of WssGBLUP compared to ssGBLUP**

| **Lines** | **Description** | **ADFI** | **ADG** | **BF** | **LDP** |
| --- | --- | --- | --- | --- | --- |
| ML1 | Top40k | 0.89 | 0.60 | 0.64 | 0.68 |
|  | Top40k weighted | 0.89 | 0.59 | 0.64 | 0.68 |
|  | ChipPlusSign | 0.90 | 0.56 | 0.61 | 0.66 |
|  | ChipPlusSign weighted | 0.98 | 0.56 | 0.62 | 0.66 |
| ML2 | Top40k | 0.90 | 0.73 | 0.69 | 0.77 |
|  | Top40k weighted | 0.88 | 0.72 | 0.66 | 1.10 |
|  | ChipPlusSign | 0.98 | 0.74 | 0.64 | 0.77 |
|  | ChipPlusSign weighted | 0.99 | 0.74 | 0.63 | 1.10 |
| TL1 | Top40k | 0.68 | 0.64 | 0.72 | 0.69 |
|  | Top40k weighted | 0.69 | 0.64 | 0.72 | 0.69 |
|  | ChipPlusSign | 0.74 | 0.63 | 0.70 | 0.70 |
|  | ChipPlusSign weighted | 0.74 | 0.62 | 0.71 | 0.70 |
| TL4 | Top40k | 0.86 | 0.65 | 0.65 | 0.71 |
|  | Top40k weighted | 0.79 | 0.65 | 0.65 | 0.71 |
|  | ChipPlusSign | 0.80 | 0.66 | 0.64 | 0.71 |
|  | ChipPlusSign weighted | 0.84 | 0.64 | 0.63 | 0.70 |
